# High prevalence of urinary schistosomiasis in a desert population: results from an exploratory study around the Ounianga lakes in Chad

**DOI:** 10.1186/s40249-021-00930-4

**Published:** 2022-01-07

**Authors:** Wendelin Moser, Annour Adoum Batil, Rebekka Ott, Moussa Abderamane, Ruth Clements, Rahel Wampfler, Sven Poppert, Peter Steinmann, Fiona Allan, Helena Greter

**Affiliations:** 1 Swiss Tropical and Public Health Institute, Basel, Switzerland; 2 University of Basel, Basel, Switzerland; 3 Institut de Recherche en Elevage Pour le Développement, Ndjamena, Chad; 4 Geology Department, University of Ndjamena, Ndjamena, Chad; 5 Department of Life Sciences, Natural History Museum, London, UK

**Keywords:** *Bulinus truncatus*, Chad, Malacology, Ounianga, POC-CCA, Prevalence, Sahara, *Schistosoma bovis*, *Schistosoma haematobium*, Schistosomiasis

## Abstract

**Background:**

Researching a water-borne disease in the middle of the Sahara desert might not seem the most relevant concern. However, nomadic Sahelian pastoralists health concerns regarding their livestock and anecdotal reports about trematode infections of *Fasciola* spp. and *Schistosoma* spp. in desert-raised animals justified an exploratory study focusing on the lakes of Ounianga in Northern Chad. The aim was to test whether trematode parasites such as *Schistosoma* spp. occur in human populations living around the Sahara desert lakes of Ounianga Kebir and Ounianga Serir in northern Chad.

**Methods:**

The study was carried out in January 2019 and comprised of three components. First, a cross sectional survey based on a random sample drawn from the population to detect infections with *S. haematobium* and *S. mansoni*; second, focus group discussions exploring disease priorities, access to health and health seeking behaviour; and third, surveying water contact sites for intermediate host snails. Samples of trematode parasites and snails were confirmed on species level by molecular genetic methods. For parasitological and malacological surveys descriptive statistics were performed. Qualitative data analysis included the full review of all transcripts, followed by a descriptive and explorative thematic analysis.

**Results:**

Among 258 participants, the overall *S. haematobium* prevalence using urine filtration was 39.2% [95% confidence interval (*CI*): 33.5–45.1%], with 51.5% of the infected suffering from heavy infection. The intermediate host snail of *S. haematobium* (*Bulinus truncatus*) occurred at water contact sites near both study villages, revealing the potential for local transmission. Although a positive *S. mansoni* point-of-care circulating cathodic antigen (POC-CCA) test result was obtained from 8.6% (95% *CI* 5.7–12.8%) of the samples, no intermediate host snails of *S. mansoni* were found, and the relevance of *S. mansoni* remains uncertain. Qualitative findings underline the importance of morbidity caused by urinary schistosomiasis, and the lack of access to diagnostics and treatment as a major health concern.

**Conclusions:**

This research revealed a high prevalence of urinary schistosomiasis in the population living around the lakes of Ounianga in the Sahara, a United Nations Educational, Scientific and Cultural Organization (UNESCO) world heritage site in Chad. Despite the high public health importance of the associated morbidity expressed by the population, there is no access to diagnostics and treatment. Further work is needed to develop and test a context-adapted intervention.

**Graphical Abstract:**

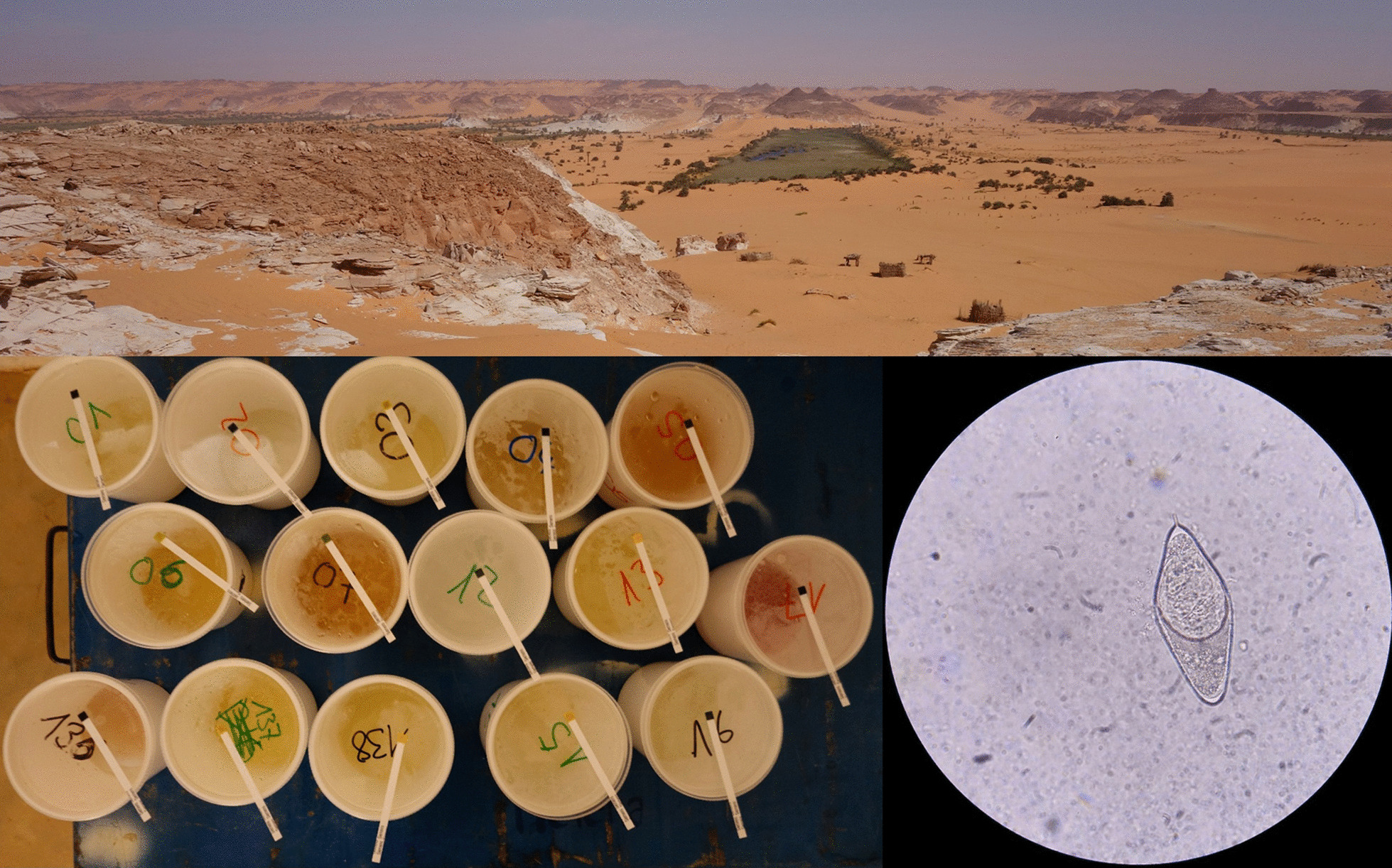

**Supplementary Information:**

The online version contains supplementary material available at 10.1186/s40249-021-00930-4.

## Background

Schistosome infections are listed among the 20 neglected tropical diseases (NTDs) targeted by the World Health Organisation (WHO) for elimination by 2030 [1]. In endemic regions, populations affected by schistosomiasis are often those living in poverty and/or in settings with restricted access to clean water for their sanitation and hygiene needs [2]. Worldwide, an estimated 230 million people harbour an infection with *Schistosoma* spp [3]. Occupational and recreational activities in close contact with freshwater, e.g., fishing, doing laundry and bathing present the main risk of infections. Highest prevalences are commonly observed among school-aged children as they enjoy playing in stagnant water sites. Undetected, and therewith untreated, urinary or intestinal schistosomiasis leads to chronic infections and serious morbidities including a wide range of different pathologies, e.g. anaemia, stunted growth, impaired cognition and organ damages, that negatively affect economic activities and therewith maintain poverty [4, 5]. A safe and effective drug, namely praziquantel, is currently used for mass drug administration programs in endemic settings as well as for treatment of individual acute infections. However, its wide use in mass drug administration programs since 2006 bears the risk of selecting for resistance in the parasite population, a topic that raises concerns when therapeutic failure is observed and that is closely monitored [6].

The majority of schistosomiasis cases occur in sub-Saharan Africa, and the disease is also reported from countries throughout the Sahel, including Mauretania, Mali, Niger, Chad and Sudan [7–11]. Infections in the Sahel ecoregion are predominantly due to *Schistosoma haematobium* which has the ability to maintain its life cycle in a semi-arid environment [12]. Yet, there are old reports on schistosomiasis occurrence from within the Sahara desert [7, 13]. The occurrence of *Schistosoma* spp. in the hot and hyper-arid desert may seem surprising but endemicity in at least two desert-specific ecosystems has been described so far. These are (a) oases where schistosome transmission is linked to man-made irrigation systems [14, 15], and (b) areas with reclaimed land for agriculture, made cultivable by artificial irrigation from deep wells [16].

Anecdotal reports from nomadic Sahelian pastoralists on *Fasciola* spp., another digenean trematode species, in livestock raised in the Chadian Sahara and recent reports about modern and early Holocene finding of intermediate host snails pointed towards the occurrence and potential ongoing transmission of schistosomiasis at the desert lakes of Ounianga, Chad [17]. Triggered by these information, an exploratory study was conceptualized with the aim to investigate whether trematode parasites such as *Schistosoma* spp. occur in two settlements at the lakes of Ounianga, Ennedi Ouest province, in Northern Chad. The study was covering three aspects, namely parasitology, malacology and the population’s health priorities, their access to health care and treatment.

## Methods

### Study site and study population

**Fig. 1:**
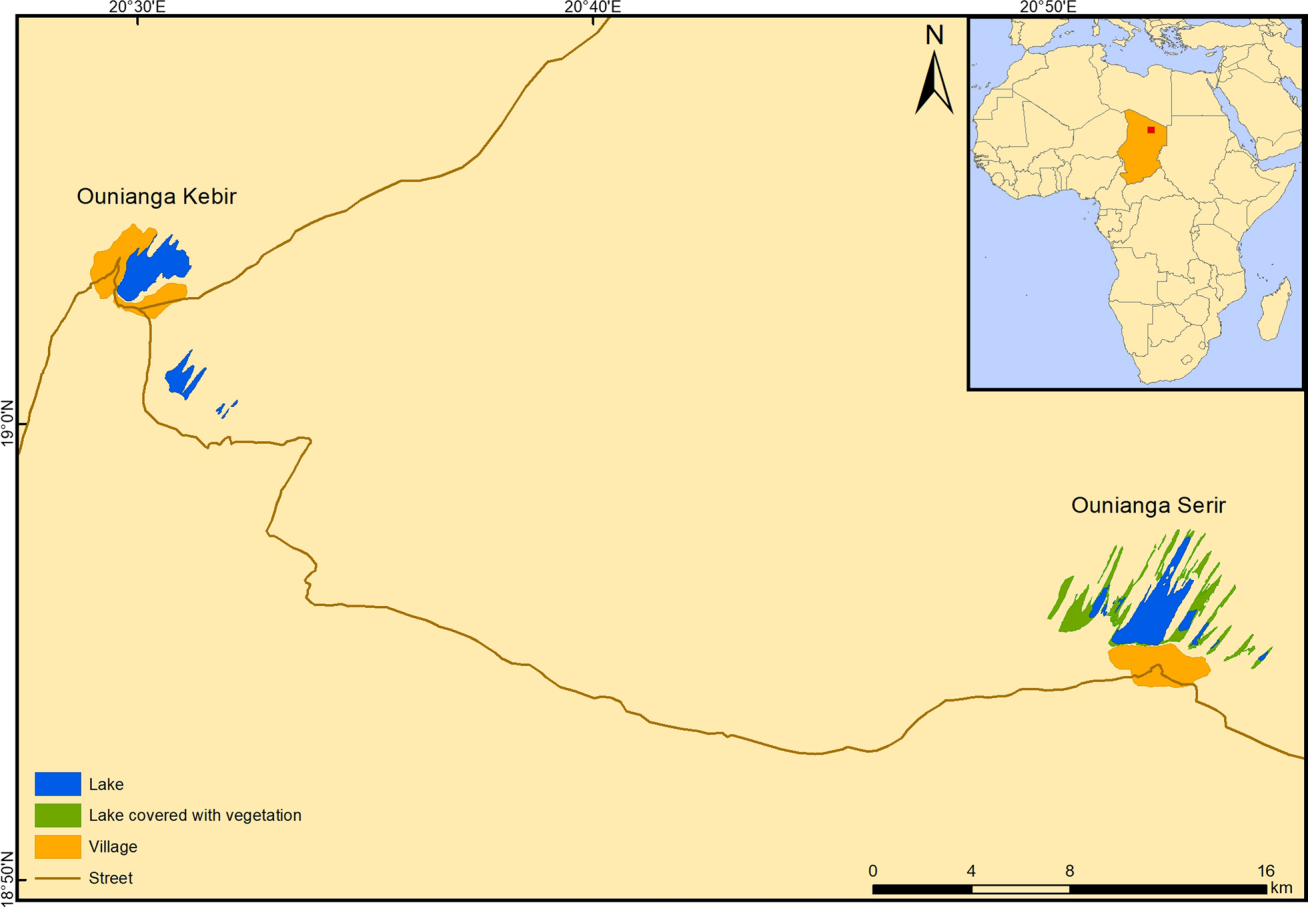
A map showing the lakes and the settlements of Ounianga Kebir and Ounianga Serir in Northern Chad

The study was carried out in January 2019 around the lakes of Ounianga and in the two settlements of Ounianga Kebir and Ounianga Serir, Ennedi Ouest province in Northern Chad (Fig. 1). The lakes lie around 40 km apart from each other within the hyper-arid Sahara desert with high daytime temperatures and less than 5 mm of rainfall per year. The lakes are fed by an underground aquifer, thereby maintaining the fresh water in some of the lakes despite the enormously high evaporation rates. Therewith, the lakes of Ounianga represent a unique hydrological system [18].

The official population estimates according to the latest national population census for Ounianga Kebir stand at around 9000 people and for Ounianga Serir at about 1000 people [19]. The majority of these people belong to one of the three predominant ethnolinguistic groups of the Tedega, Dazaga Toubou and the Zaghawa. Their main occupational activities include pastoralism, natron mining and trade. In both communities, the primary schools are operational, yet not the secondary schools. The only functional health centre of the Ounianga district is located in Ounianga Kebir and its catchment population is estimated to include 30,000 people. Ounianga Serir has no functional health centre; the population has set up a health post to provide basic health services to the community members.

### Parasitological survey

The resident population of Ounianga Kebir and Ounianga Serir, older than 5 years of age, were eligible for participation. The sample size was calculated using Epi Info 7.1.3.3 (Centre for Disease Control (CDC), Atlanta, GA 30333, USA). Parameters used were “population survey” with two-sided confidence intervals of 95%, an expected frequency of 50% and a population size of 10,000, resulting in a sample size of 370. Proportional to the total population estimates, the targeted sample size repartition was 330 people in Ounianga Kebir and 40 people in Ounianga Serir. At household level and at the primary schools, individuals were randomly selected by applying the spatial sampling method from the Expanded Programme on Immunization (EPI) of the WHO as previously published [20]. After obtaining oral consent from each selected individual, or in case of children from their caretakers, they were asked to produce a urine sample around noon, when *S. haematobium* egg excretion is known to peak [21]. A mobile field laboratory was set up at the health centre, and health post, respectively. The urine samples were analysed for haematuria by reagent strip testing (Hemastix; Siemens Healthcare Diagnostics GmbH; Eschborn, Germany) and classified as negative, light and severe haematuria as outlined by the testing handbook. Subsequently, 10 ml samples were subjected to urine filtration using a syringe and pressed through a 13-mm diameter filter holder containing a 20-μm wire-mesh filter (Sefar AG; Heiden, Switzerland), followed by microscopic screening of the filter content for the presence of *S. haematobium* eggs. The infection intensity was determined according to the 3 categories defined by the WHO as no infection (0 egg/10 ml urine), mild infection (1–49 eggs/10 ml urine) and heavy infection (50 or more eggs/10 ml urine) [22]. A point-of-care circulating cathodic antigen (POC-CCA) urine cassette test (Rapid Medical Diagnostics; Pretoria, South Africa) was performed to screen for *S. mansoni* infections. The POC-CCA test applies a lateral flow principle and allows to detect adult *Schistosoma* worm CCA in the hosts’ urine by adding a colloid carbon conjugate of a monoclonal antibody to the sample, and has been validated before for its performance under extreme environmental conditions as those occurring in the Sahara [23, 24].

### Qualitative survey

In both communities, one focus group discussion (FGDs) with men and one with women were organized. Additionally, one FGD was organized with the staff of the health centre in Ounianga Kebir. In Ounianga Serir, an in-depth interview (IDI) was carried out with the person responsible for the health post. The topics covered by the interview guides were disease priorities and priority health issues, perceptions and health seeking behaviour. Both FGDs and IDIs were assisted by an interpreter who translated the conversation from Arabic to French, allowing the study team to take notes. Digital recordings of the FGDs and IDI were transcribed and translated into French, integrating the notes taken during the FGDs or IDIs.

### Malacological survey

At both settlements, individual community members and school-aged children were asked to guide the team to all frequented human-water contact sites. At each site, GPS coordinates and the water parameters temperature (°C), pH, conductivity (µs/cm) and dissolved oxygen (mg/L) were recorded, using a portable multimeter (Hach^®^, HQ40D, Loveland, USA). For turbidity, a turbidimeter was used [Formazin Nephelometric Units (FNU); Hach^®^, 2100P Iso]. The snail sampling was performed adhering to standard protocols. In short, for 15 min, all aquatic snails were collected by one person using a scoop or forceps to detach them from aquatic and subaquatic plants [25]. Subsequently, the snails were placed on wet cotton in petri dishes, and transferred to the field laboratory. Snails were identified to the genus or, if possible to species level on site. At midday, each collected snail identified as intermediate host species was placed in a well plate filled with bottled drinking water and exposed to daylight for three hours to induce cercarial shedding [26]. The snail size (in mm) and weight (in mg) was measured using a calibre and balance, respectively. Thereafter, all snail specimens were conserved in 70% ethanol, and shipped to the National History Museum, London (NHM) for molecular analysis.

### Snail species confirmation using molecular methods

The snail samples selected for the molecular analyses represented individuals from each collection site. On arrival at the NHM, the snail species identification was confirmed based on morphological characters and samples re-spirited (absolute ethanol) for incorporation into the Schistosomiasis Collection at the Natural History Museum (SCAN) [27]. Photographic images were taken of the snail shells prior to DNA extraction. Specimens were placed in TE buffer (10 mmol/L Tris, 0.1 mmol/L EDTA) pH 7.4 for one hour in order to remove any remaining ethanol from within the tissue, which might interfere with subsequent extraction steps [28]. Total genomic DNA was isolated from head/foot tissue using the DNeasy Blood and Tissue kit (Qiagen, UK) according to manufacturer’s instructions. DNA was eluted into 200 μl sterile water.

### Amplification of *Cox1* fragments of snail DNA

A polymerase chain reaction (PCR) amplification of a partial cytochrome oxidase 1 (*Cox1*) sequence was performed using primers LCO1490 (5′-GGTCAACAAATCATAAAG ATATTGG-3′ forward) and HCO2198 (5′-TAAACTTCAGGGTGACCAAAAAATCA-3′ reverse) [29]. PCR investigations and sequencing conditions were chosen as previously outlined [28, 30].

### Checking of sequence data

The electropherograms produced were checked and *Cox1* sequences edited using Geneious, version 11.0.5 (http://www.geneious.com) [31]. Sequences were compared to database entries by performing BLAST searches via the National Center for Biotechnology Information against GenBank and EMBL sequence databases; and aligned with reference material.

### Sequencing of *Schistosoma* spp. eggs in urine

Positive urine samples from Ouinanga Kebir were combined into seven different pools of 8–12 ml respectively one pooled sample of 12 ml for the villages of Ouinanga Serir. Samples were shipped to the diagnostic centre of the Swiss Tropical and Public Health Institute (Swiss TPH) in Basel, Switzerland for further processing. There, each pool was centrifuged at 3000 × *g* for 10 min. Exactly 500 µl of the pellet was re-suspended and transferred to a 2 ml tube containing garnet beads. After addition of 1 ml PBS, the sample was centrifuged 1 min at 13,000 × *g* and the supernatant was discarded. The pellet with the garnet beads was frozen 30 min at −80 °C and further processed as described by Barda and colleagues [32, 33]. Samples were first tested by simplex generic *Schistosoma* spp. 28S real-time PCR amplifying *S. mansoni*, *S. haematobium*, *S. intercalatum*, *S. bovis* [34] and additionally *S. japonicum* because of modifications added to the second reverse primer of the assay (Additional file 1: Table S1). The reaction mix contained 1 × *Taq*Man GenExpression MasterMix (ThermoFisher Scientific, Basel, Switzerland), 800 nmol of forward primer, 400 nmol of each reverse primer and 200 nmol of probe. The samples were subsequently tested by a duplex real-time PCR for the presence of a specific *S. mansoni* TRE region and of *S. haematobium* dra1 sequence [34, 35]. Each reaction mix contained 1 × *Taq*Man GenExpression MasterMix (ThermoFisher Scientific, Basel, Switzerland), 800 nmol of each primer, 200 nmol each probe (Additional file 1: Table S1). The thermoprofile of all assays on the QuantStudio5 (ThermoFisher) consisted of 2 min at 50 °C, 10 min at 95 °C followed by 45 cycles of 15 s at 95 °C and 1 min at 58 °C. The specificity of all assays was previously tested on a variety of DNA from stool and blood samples including: *Ascaris lumbricoides, Blastocystis hominis, Cryptosporidium spp., Dientamoeba fragilis, Encephalitozoon spp., Endolimax nana, Entamoeba coli, E. dispar, E. histolytica, E. moshkovskii, E. polecki, Enterocytozoon bieneusi, Giardia lamblia, Hymenolepis nana, Iodamoeba bütschlii, Sarcocystis* spp.*, Taenia* spp*., Strongyloides stercoralis, Trichuris trichiura, Plasmodium falciparum, P. vivax, P. malariae, P. ovale, Trypanosoma cruzi, T. brucei, Leishmania* spp. and was found to be 100% specific. Analytical limit of detection (LOD) was tested by a plasmid dilution row ranging from 10^7^ to 10^–1^ plasmids/µl containing an insert with the sequence of the *Schistosoma* real-time PCR product, and was found to be at 10 plasmids/µl for all assays. On each real-time PCR plate and for each target we included negative and positive low-copy plasmid controls.

Subsequently, all samples were tested by classic PCR of the *COX* gene of *S. haematobium* and *S. bovis* as modified from Boon and co-workers (Additional file 1: Table S1) [36]*.* The reaction mix contained 1 × HotStar*Taq* Plus Master Mix (Qiagen, Hilden, Germany), 800 nmol of each primer, 5 µl DNA in a total reaction volume of 50 µl. The thermoprofile consisted of 5 min at 94 °C followed by 40 cycles of 40 s at 94 °C, 40 s at 58 °C and 1 min at 72 °C and a final step of 10 min at 72 °C. After visualization on a 2% agarose-gel, the positive sample of the *S. bovis-*COX PCR was sent for Sanger sequencing with the primers of amplification at Microsynth AG (Baldach, Switzerland). The sequence was then compared to database entries by performing BLAST searches via the National Center for Biotechnology Information. The sequence is accessible in GenBank under the number: MW937895. A table listing primers and probes is accessible in the supplementary information (Additional file 1: Table S1).

### Statistical analysis

Descriptive statistics of parasitological and malacological data was performed using STATA version 16.0 (STATA Corp Inc., TX, USA) and ArcGIS (Version 10.7.1.; ESRI Inc. ArcMap™ 10.7, Redlands, CA, USA). Qualitative data analysis included the full review of all transcripts, followed by a descriptive and explorative thematic analysis.

## Results

### Parasitological survey

In both study sites, urinary schistosomiasis was highly prevalent. Indeed, 35.3% (95% *CI* 29.1–41.9%) of the tested participants were *S. haematobium* egg positive in Ounianga Kebir and 54.9% (95% *CI* 40.8–68.2%) in Ounianga Serir, resulting in an overall prevalence of 39.2% (95% *CI* 33.5–45.1%) (Table 1). The *S. haematobium* prevalence was highest among children and adolescents below 18 years of age in both villages. In Ounianga Kebir, more boys than girls were infected (51.1% *versus* 36.9%), whereas in Ounianga Serir girls had a higher prevalence (72.7%).

**Table 1:** Prevalence of *S. haematobium* infection by egg positivity and infection intensities, haematuria and *S. mansoni* test results from POC-CCA testing (trace results excluded) in the study population from Ounianga Serir and Ounianga Kebir, Chad, 2019

	Ounianga Kebir (*n* = 207)		Ounianga Serir (*n* = 51)		Total *n* (%, 95% *CI*)
	Male *n* (%, 95% *CI*)	Female *n* (%, 95% *CI*)	Male *n* (%, 95% *CI*)	Female *n* (%, 95% *CI*)	
Total number of participants	65	142	21	30	258
Participants aged < 18 years	47 (72)	65 (46)	14 (67)	11 (37)	137 (53)
*S. haematobium* infection (egg positive)					
Total no. positive	27 (41.5, 30.0–54.0)	46 (32.4, 25.2–40.5)	11 (52.4, 30.2–73.6)	17 (56.7, 37.9–73.7)	101 (39.2, 33.5–45.1)
Positives < 18 years*	24 (51.1, 36.6–65.3)	24 (36.9, 25.9–49.5)	9 (64.3, 34.1–86.3)	8 (72.7, 35.4–92.8)	65 (47.5, 39.2–55.8)
*S. haematobium* heavy infection (≥ 50 eggs/10 ml urine)					
Total no. heavy infection	13 (50.0, 30.5–69.5)	21 (45.7, 31.6–60.5)	8 (72.7, 35.4–92.8)	9 (52.9, 28.1–76.4)	51 (51.0, 41.2–60.7)
Heavy infection < 18 years	13 (56.5, 34.8–76.0)	14 (58.3, 36.9–77.0)	8 (88.9, 37.5–99.1)	7 (87.5, 32.0–99.1)	42 (65.6, 53.0–76.4)
Haematuria					
Total no. positive	36 (55.4, 43.0–67.2)	82 (57.8, 49.5–65.6)	12 (57.1, 34.2–77.4)	22 (73.3, 53.9–86.6)	152 (58.9, 52.9–64.6)
Positives < 18 years*	26 (55.3, 40.6–69.2)	39 (60.0, 47.5–71.3)	9 (64.3, 34.1–86.3)	9 (81.8, 42.1–96.5)	83 (60.6, 52.2–68.4)
*S. mansoni* infection (from POC-CCA test results)					
Total no. positive	3 (4.7, 1.5–13.8)	11 (8.4, 4.7–14.5)	5 (23.8, 9.5–48.2)	2 (7.1, 1.6–26.2)	21 (8.6, 5.7–12.8)
Positives < 18 years*	3 (6.5, 2.0–19.0)	5 (8.3, 3.4–18.8)	5 (35.7, 13.7–65.9)	1 (9.1, 0.9–53.6)	14 (10.7, 6.4–17.2)

*(%) from all participants < 18 years of age; POC-CCA: Point-of-care circulating cathodic antigen

**Fig. 2:**
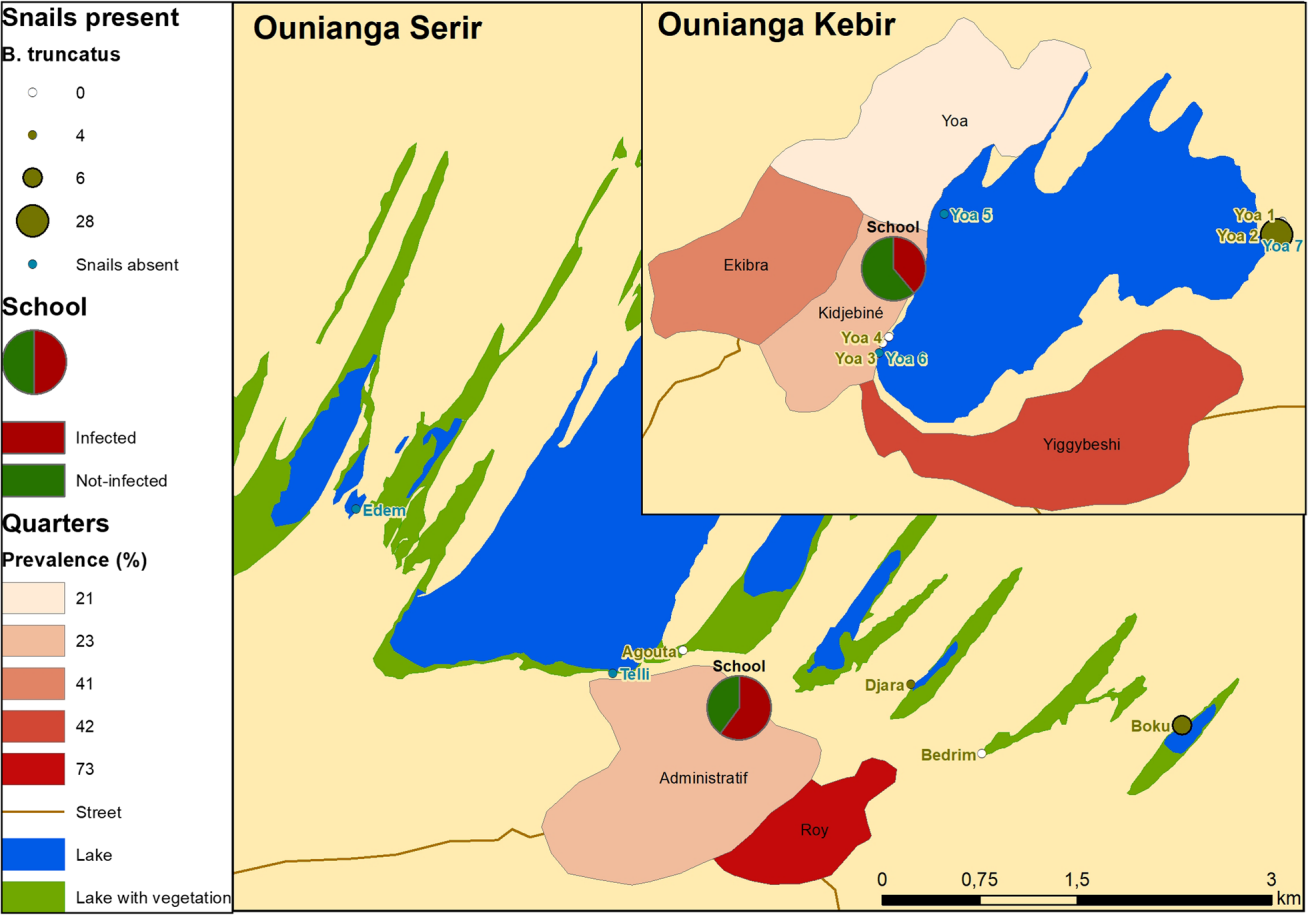
Map showing the urinary schistosomiasis prevalence and snail abundance for Ounianga Kebir and Serir. The prevalence of urinary schistosomiasis among participants is stratified by neighbourhood. For each water site sampled, the abundance of the intermediate host snail *Bulinus truncatus* is indicated

Mapping of the schistosomiasis prevalence by place of living (neighbourhood) shows a slightly higher prevalence for those neighbourhoods closer to a water site where the aquatic intermediate host snail *Bulinus truncates* was present (Fig. 2, Ounianga Kebir: Yiggybeshi, Ounianga Serir: Roy). Regarding the POC-CCA testing for *S. mansoni*, 8.6% of the urine samples showed a positive test results.

**Fig. 3:**
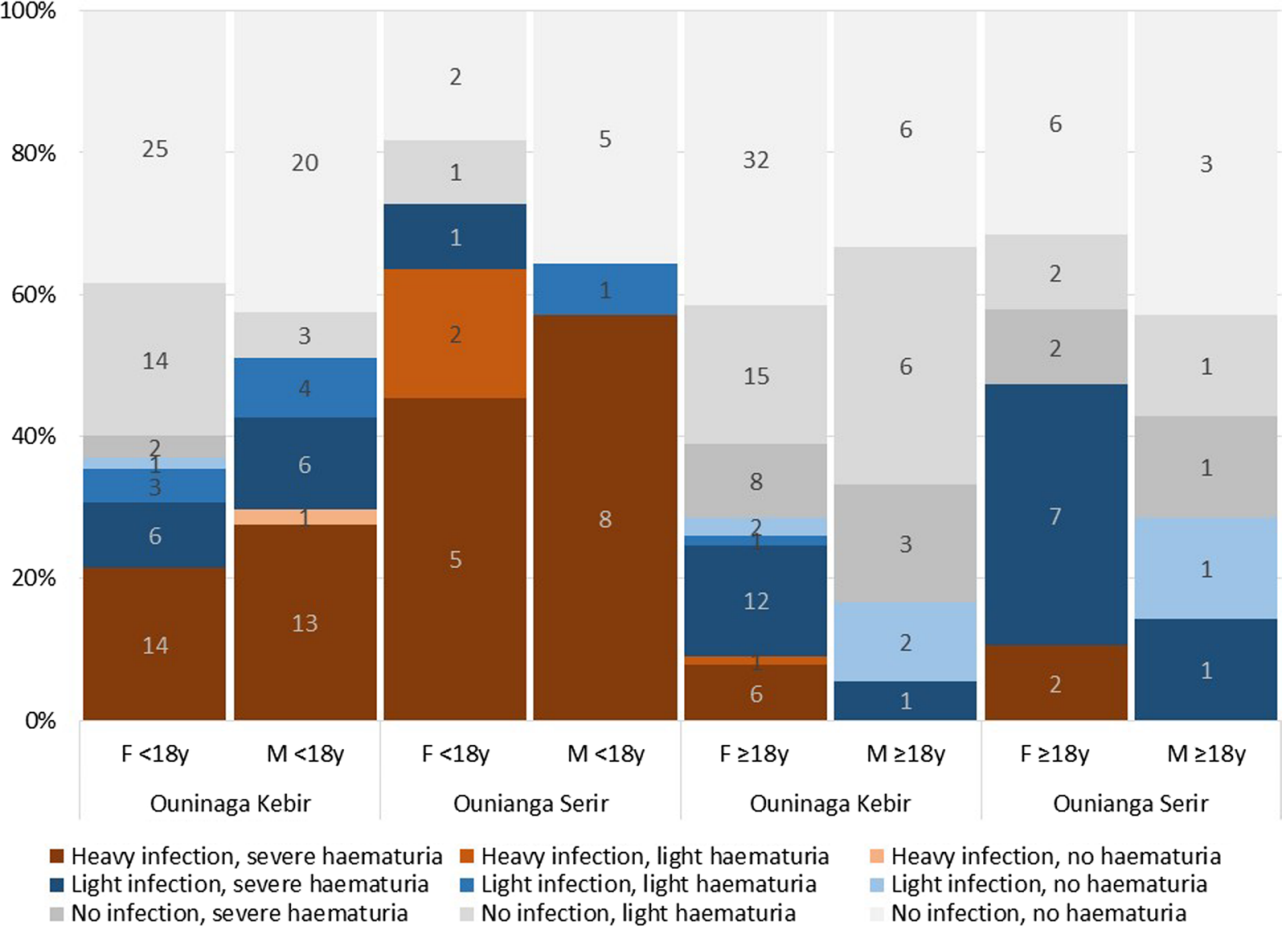
Schistosomiasis infection intensity and haematuria stratified by sex, age group and place of living

More than half of all infected participants harboured a heavy *S. haematobium* infection (51.0%; heavy infection: > 50 eggs/10 ml urine) and the burden was higher in children, whereof over two third were heavily infected (Table 1) [37]. Overall, 10.2% of all egg-negative, 69.4% of all light and 92.3% of all heavily infected participants had severe haematuria, with no big differences between age groups and gender (Fig. 3).

### Qualitative survey

During FGDs in both study sites, abdominal issues and blood in urine were the most frequently mentioned health problems among adults and also in children. Health staff mentioned that the majority of patients seeking care at the centre for any cause additionally suffers from abdominal issues. Among children, diarrhoeal diseases, respiratory infections and scorpion stings were reported as major health issues. Adults also suffered from eye problems, headache and joint pain. Fertility issues are another major concern and women reported difficulties getting pregnant again after they had their second or third child.

A major constraint for people in both sites is the difficult access to health facilities. Accessible facilities are usually underequipped; rarely have drugs available and the personnel had only basic training. To obtain appropriate care and treatment, people needed to travel long distances within Chad (to Faya, Abeche and Ndjamena) or abroad to Libya or Sudan.

Another common theme was the lack of safe drinking water as pumps are rare and open wells are commonly used as water sources. Perceived water quality is low due to salty taste and visible contamination.

The population was well aware of parasitic diseases, yet had limited knowledge on risk factors and transmission. Blood in urine was linked to parasitic infections, low quality of drinking water, water contact at the nearby lakes, or kidney issues. *Kadi* and *Kouli* are two local names for parasite infections linked to abdominal pain. *Kadi* describes an intestinal worm infection causing symptoms like intestinal spasms and flatulence, increased appetite with the tendency of weight loss. As a traditional treatment, infected people are given natron, a naturally occurring form of sodium bicarbonate, or an extract of the roots of a plant called *Boa* to initiate diarrhoea, causing a worm with a red ‘mouth’ to leave the body via the excrements. The symptoms described for *Kouli* correlate with symptoms of the parasite *Enterobius vermicularis* such as persistent itching in the perianal area and sleep disturbances. The traditional treatment administered to *Kouli* patients are eating butter or drinking an extract of a medical plant called *Chi*.

*Ouco* is the local term to describe the health condition related to blood in urine combined with pain while urinating and reduced male erectile function. In traditional medicine, the urine of the animal called *Nii* (Fennec Fox, *Vulpes zerda*) is believed to have a curative effect.

The reported level of satisfaction with access to medical treatment for the above mentioned health issues is mixed. Important challenges mentioned included stock outs of medicines, lack of diagnostic means and non-effectiveness of the medical treatment received. Especially the female FGD participants expressed a need for health education and sensitization among the population.

### Malacological survey

Among a total of 17 different collection sites, 8 harboured freshwater snails (Table 2, Fig. 2). The highest numbers of snails were collected from the two intermediate host snail species *Radix natalensis* (*n* = 42) and *Bulinus truncatus* (*n* = 38), and four species of no medical importance (*n* = 42). Snails were identified using shell shape and a partial *COX*1 sequence. Particularly high numbers of any snail species were collected at two sites, namely Yoa 2 (*n* = 35) in Ounianga Kebir and Agouta (*n* = 34) in Ounianga Kebir. Among all snails, only one *B. truncatus* was shedding cercariae (from Yoa 2). Upon testing, they were recognized to not represent *S. haematobium* cercariae, and consequently were not further studied. The average shell height of all *R. natalensis* specimen was 10.6 mm (95% *CI*: 9.08–12.11 mm), and 6.66 mm (95% *CI*: 5.97–7.34 mm) for *B. truncatus*,.

**Table 2:** Snail abundance and water parameters for each sampling site

Site*		Snail species	No. of snails found	Temperature (°C)	Conductivity (µs/cm)	pH	Oxygen (mg/L)	Turbidity (FNU)
Sampling sites where snails were present								
Ounianga Kebir								
Yoa (Girki)	1*	*No host snail species*	0	17.5	1054.0	6.8	2.7	1.3
	2*	*B. truncatus*	28	14.5	1046.0	6.9	4.1	2.5
		*R. natalensis*	6					
Yoa (source 2)	3*	*R. natalensis*	19	22.7	1941.0	7.1	3.2	1.9
	4*	*R. natalensis*	7	22.8	2.56	7.0	3.0	3.7
Ounianga Serir								
Agouta		*R. natalensis*	7	14.5	8.0	8.3	4.9	3.5
Djara		*B. truncatus*	4	19.7	6.0	8.5	6.6	2.4
Boku		*B. truncatus*	6	20.8	1524.0	8.8	8.8	5.9
		*R. natalensis*	3					
Bedrim		*No host snails species*	0	18.7	7.3	8.4	5.2	3.0
Sampling sites where no snails were present								
Ounianga Kebir								
Yoa 5 (hot source)				28.4	2.8	7.0	2.9	4.9
Yoa 6 (hot source)				27.7	2107.0	7.4	3.2	16.3
Yoa 7 (lake)				14.4	290.0	10.3	1.1	158.0
Uma red				27.5	> 2000.0	10.1	14.2	85.8
Uma blue				18.6	> 2000.0	10.5	15.0	118.0
Uma (hot spring)				28.2	4.0	9.1	3.6	14.9
Forodone				19.1	31.1	9.8	2.0	> 1000.0
Ounianga Serir								
Telli				21.3	13.7	10.7	2.7	4.5
Edem				14.1	3.5	8.7	8.3	1.0

*All sampling sites are shown in Fig. 2

Across all sites where snails were found, the average water temperatures was 18.9 °C (standard deviation ± 3.3), the average oxygen content was 4.8 mg/L (± 2.1) and a turbidity of 3.0 FNU (± 1.4). The sites without snails were characterized by a wide range of measured water parameters, i.e., temperature of 22.1 °C (range 14.1–28.2), oxygen 5.9 mg/L (range 1.1–15.0) and turbidity 267.0 FNU (range: 1.0– > 1000.0). Snails obviously preferred the pH range between 7.0 and 8.8 compared the sites without snails with a pH varying between 7.0 and 10.5. Inconclusive results were found for the conductivity comparing sites with and without snails with a range of 6.0–1941.0 µs/cm and 2.8 to > 2500.0 µs/cm, respectively (Table 2).

### Sequencing of *Schistosoma spp.* eggs in urine

All eight urine pools were positive in the generic *Schistosoma* spp. 28S real-time assay, in the *S. haematobium* dra1 real-time assay and in the *S. haematobium* COX1 PCR consistent with the presence of *S. haematobium* eggs in all pools. No pool was positive for *S. mansoni* TRE real-time PCR. One pool from the village of Ouinanga Serir was positive for *S. bovis* COX1. This result indicates the possibility of the presence of *S. haematobium* X *bovis* hybrids as observed in previous studies in West Africa [36, 38].

## Discussion

According to the statements of the local authorities, this exploratory study was the very first time a medical research team visited the district of Ounianga, Ennedi Ouest province in Chad. Our research revealed a high prevalence of *S. haematobium* in the population of both Ounianga Kebir and Serir villages. Living specimens of *B. truncatus* were found at both sites, whereas the previous findings were fossils dating back to the early Holocene [17]. These findings strongly suggest ongoing local schistosome transmission in this desert oasis environment.

The larger of the two villages, Ounianga Kebir, had an overall lower schistosomiasis prevalence compared to the smaller village of Ounianga Serir (35.3% versus 54.9%). In the different neighbourhoods of Ounianga Kebir the prevalence varied and ranged from 21 to 42% (Fig. 2). This may be partly explained by the proximity to the rare freshwater sites that are used for washing cloth, bathing and swimming. For example, Lake Yoa with its cold temperature and high salinity is fed by numerous freshwater springs. These provide habitats for the intermediate host snails, and the neighbourhood with the highest prevalence was the one closest to a freshwater spring. The quarters with lower prevalence were closer to freshwater sources including the two hot springs (Yoa 5 and 6). Here, the high water temperature might explain the absence of snails [39]. The two adjacent sampling sites Yoa 3 and 4 are cold and only used for irrigating the surrounding gardens or for watering livestock. Interestingly, at these two sites snails of the species *Radix natalesis* were found, the intermediate host of the liver fluke *Fasciola* spp. In Ounianga Serir, there are no hot springs and the two freshwater lakes (Djara and Boku) are used for all water-related activities. In both, *B. truncatus* were present and the lakes’ close proximity to the quarter Roy may explain the high urinary schistosomiasis prevalence. The snails were identified using shell morphology and molecular methods; diversity within species was low, however it would be of interest to sequence further with other genes such as ITS. A single snail was found to be shedding cercariae but not *S. haematobium.* To inform on where transmission could be occurring, additional testing of the snails for prepatent infections will be carried out.

About half of the adult participants and two thirds of the children with a positive parasitological test suffered from heavy *S. haematobium* infections. Our data show that the infection intensity is associated with the severity of haematuria, pointing towards chronic schistosomiasis caused by long-term exposure and recurrent reinfection. Hence, the major health problems reported by the local population, namely abdominal issues and blood in urine, may well be due to schistosomiasis, and are likely the consequences of the lacking access to diagnostic and treatment options, and the absence of any preventive intervention. Similar observations have also been reported from other remote areas in Chad [40].

The study was designed as an exploratory study with the aim to reveal the presence of the full *Schistosoma* spp. lifecycle in these desert lakes. Its scope was therefore limited and findings leave several factors unaddressed at this stage. For example, the men’s main activities involve working in natron extraction sites, trading using traditional caravans, and raising livestock through mobile pastoralism. Hence, during the visit of the study team, the majority of men aged 16–60 years were absent, resulting in an over-representation of women in the study population (ratio 1:2). Regarding the *S. mansoni* diagnostics that showed a positive POC-CCA result for 8.6% of all urine samples, we cannot conclude with certainty that *S. mansoni* is present in the study population as no stool samples were collected and hence, no parasitological proof of *S. mansoni* infection is available. Of note, according to the test handbook, also a heavy infection with *S. haematobium* can lead to a positive test result [24]. It is also significant that no intermediate host snails of the genus *Biomphalaria* were found*.* However, the exploratory study was conducted in January while snail abundance is highly seasonal [26].

## Conclusions

This exploratory study presents the first modern evidence of urinary schistosomiasis among the population of these oasis villages. A high prevalence of about 40% of urinary schistomiasis was found, with no access to diagnostics and effective treatment with praziquantel. The presence of intermediate host snails in this isolated area suggests ongoing local transmission. There is clearly a need for further studies to fully understand the current epidemiological situation. However, apart from further studies the main problems are already evident; namely the lack of health education, diagnostics and access to treatment. With a combined approach, including sensitization, mass drug administration, and morbidity management the control or even elimination of urinary schistosomiasis in this population might be possible.

## Supplementary Information

**Additional file 1.** List of primers and probes used for sequencing of *Schistosoma* spp. eggs.

## Data Availability

Data will be available on request by email to the corresponding author.

## Abbreviations

BASEC: Business Administration System for Ethics Committees
CDC: Centre for Disease Control
*CI*: Confidence interval
CNBT: Comité National de Bioéthique du Tchad
EPI: Expanded Programme on Immunization
FGD: Focus group discussion
FNU: Formazin nephelometric units
IDI: In-depth interview
LOD: Analytical limit of detection
NHM: Natural History Museum
NTD: Neglected tropical disease
POC-CCA: Point-of-care circulating cathodic antigen
SCAN: Schistosomiasis Collection at the Natural History Museum
UNESCO: United Nations Educational, Scientific and Cultural Organisation
WHO: World Health Organization
