# Supplementary material for: High prevalence of urinary schistosomiasis in a desert population: results from an exploratory study around the Ounianga lakes in Chad
Source: Infect Dis Poverty. 2022 Jan 7;11:5. doi: 10.1186/s40249-021-00930-4 (PMC8740043; doi:10.1186/s40249-021-00930-4)
Supplement: Supplementary file 1 — Additional file 1. List of primers and probes used for sequencing of Schistosoma spp. eggs. [file 40249_2021_930_MOESM1_ESM.docx]

SUPPLEMENTARY INFORMATION

**Table S1. Primers and probes**

| **Parasite** | **Name** | **Sequence 3’-5’** | **Origin** |
| --- | --- | --- | --- |
| Generic *Schistosoma spp.* | Schisto28S_F | GTGGAGTTGAACTGCAAGC | Modified from Cnops et al. 2012 |
|  | Schisto28S_R1 | CCATAGCAGACAGGCAGC |  |
|  | Schisto28S_R2 | GCTCAACAWTAATAGTCAAACCTG |  |
|  | Schisto28S_P | FAM-ACTGACAAGCAGACCCTCACACC-BHQl |  |
| *S. mansoni TRE* | Sman_F | CCACGCTCTCGCAAATAATCTA | Modified from Wichmann et al. 2013 |
|  | Sman_R | AAATCGTTGTATCTCCGAAACCA |  |
|  | Sman_P | YYE-ACAAACATCATAAAAATCCGTCCA-MGB-Q5 |  |
| *S. haematobium dra1* | Shae_F | GATCTCACCTATCAGACGAAAC | Identical to Cnops et al. 2013 |
|  | Shae_R | TCACAACGATACGACCAAC |  |
|  | Shae_P | FAM- TGTTGGTGGAAGTGCCTGTTTCGCAA -BHQ1 |  |
| *S. haematobium*  COX | SH_COX_F | TTTTTTGGTCATCCAGAGGTGTAT | Modified from Boon et al. 2018 |
|  | SH_COX_R | TAATAATCAATGACCCTGCAATAA |  |
| *S. bovis* COX | SB_COX_F | TTTTTTGGGCATCCGGAGGTGTAT |  |
|  | SB_COX_R | CACAGGATCAGACAAACGAGTACC |  |
